# Association of dietary quality and dietary inflammatory potential with inflammatory markers: evidence from the national health and nutrition examination survey 2009-2018

**DOI:** 10.3389/fimmu.2025.1596806

**Published:** 2025-06-05

**Authors:** Sida Wang, Yujin Bao, Linning Wang, Xiaoxi Xie, Yun Lu

**Affiliations:** School of International Pharmaceutical Business, China Pharmaceutical University, Nanjing, Jiangsu, China

**Keywords:** NHANES, inflammatory markers, healthy eating index, dietary inflammation index, dietary pattern

## Abstract

**Objectives:**

This study aimed to investigate the independent and joint association of dietary quality and dietary inflammatory potential with four inflammatory markers among U.S. adults and to analyze the moderating role of age.

**Methods:**

This study included 19,110 participants from the National Health and Nutrition Examination Survey (NHANES, 2009–2018). Dietary quality and dietary inflammatory potential were assessed using the Healthy Eating Index-2015 (HEI-2015) and the Dietary Inflammatory Index (DII), respectively, and thus classified into four dietary patterns. Inflammatory markers included white blood cell (WBC), neutrophil (Neu), neutrophil-to-lymphocyte ratio (NLR), and systemic immune-inflammation index (SII). Weighted multiple linear regression and weighted quantile sum (WQS) regression were employed to analyze the relationships between HEI-2015/DII and inflammatory markers. Joint effect and interaction analyses were conducted to explore the impacts of different dietary patterns and age.

**Results:**

HEI-2015 showed significant inverse associations with WBC, Neu, NLR, and SII, whereas DII exhibited significant positive associations with these markers. WQS analysis revealed that adequacy components in HEI-2015 such as seafood and plant proteins, and whole grains contributed most to reduced inflammation. In contrast, fiber, alcohol, and energy intake were the primary contributors to inflammatory markers in DII. Joint effect analysis demonstrated that compared to pattern 1, pattern 4 significantly reduced WBC, Neu, NLR, and SII levels. However, no significant reduction was observed in pattern 3. Additionally, age significantly strengthened the inverse associations between HEI-2015 and WBC/Neu, while weakening the positive associations between DII and SII.

**Conclusion:**

Improving dietary quality and reducing dietary inflammatory potential may help lower inflammatory biomarker levels, with age playing a critical moderating role. High-quality diets can counteract the adverse effects of pro-inflammatory diets, whereas solely anti-inflammatory diets cannot compensate for the detrimental effects of low-quality diets. The combined effect of both approaches may further enhance anti-inflammatory outcomes.

## 1 Introduction

Inflammation is an important protective mechanism of the body against injury and infection, but chronic low-grade inflammation plays a key role in the occurrence and development of various chronic diseases, including cardiovascular diseases (1–3), diabetes (4–6), neurodegenerative diseases (7), obesity (8, 9), and cancer (10–12). Chronic inflammation may increase the risk of chronic diseases in damage through various mechanisms such as continuous activation of the immune system and oxidative stress (13). It is noteworthy that optimal dietary patterns and nutrient composition can lower levels of certain inflammatory cytokines, thus having a positive regulatory effect on chronic inflammation (14), which is of great significance for preventing and improving chronic inflammation-related diseases.

A growing body of evidence has shown that specific dietary components regulate levels of inflammatory markers. For example, the intake of fruits and vegetables is negatively correlated with C-reactive protein (CRP) (15, 16). In an observational study, the relationship between alcohol intake and high-sensitivity CRP (hs-CRP) showed gender differences (J-shaped association in women and positive linear-shaped association in men), but no significant correlation was found between alcohol intake and leukocyte count (17). Furthermore, a clinical trial showed that high doses of omega-3 polyunsaturated fatty acids (PUFA) were associated with lower levels of interleukin-6 (IL-6) and tumor necrosis factor-α (TNF-α) (18). A recent clinical trial also found that patients supplemented with omega-3 PUFA showed a significant reduction in IL-8 and IL-17 levels in salivary samples after three months (19).

In fact, diet is not simply a sum of individual components, and the interactions between different food components may potentially influence inflammatory markers. Therefore, to systematically evaluate the impact of dietary patterns on inflammation, comprehensive dietary scoring tools should be utilized. The Healthy Eating Index, developed by the U.S. Department of Agriculture, is an indicator of diet quality (20), while the Dietary Inflammatory Index (DII), designed by Shivappa et al., is used to assess the inflammatory potential of diet (21). Both HEI-2015 and DII have been widely applied in dietary pattern research, with their scores reflecting overall dietary quality and inflammatory potential, and the two showing a significant negative correlation (22). Although previous studies have separately explored the association of HEI-2015 and DII with inflammatory markers (23–26), most current studies focus on analyzing the individual impact of dietary quality or inflammatory potential on inflammatory markers, with fewer studies examining their combined effect.

Although both dietary quality and dietary inflammatory potential are important factors influencing levels of inflammatory markers, their combined effect on inflammatory markers has not been fully studied. This study focuses on HEI-2015, DII, and four inflammatory markers, investigating the combined effect of HEI-2015 and DII on inflammatory markers. Meanwhile, age is treated as an important moderating variable to further analyze its moderating role in the association between dietary scores and inflammatory markers, providing a more comprehensive theoretical basis for dietary interventions.

## 2 Methods

### 2.1 Data source and study subjects

The data used in this study were obtained from the National Health and Nutrition Examination Survey (NHANES) database (27). NHANES is a nationally representative cross-sectional survey conducted by the National Center for Health Statistics (NCHS), aimed at comprehensively measuring the health and nutritional status of adults and children in the United States. The survey collects data through interviews, health exams in the mobile exam center, and laboratory tests, including demographic characteristics, health status, dietary information, behavioral characteristics, and exposure biomarkers.

**Figure 1:**
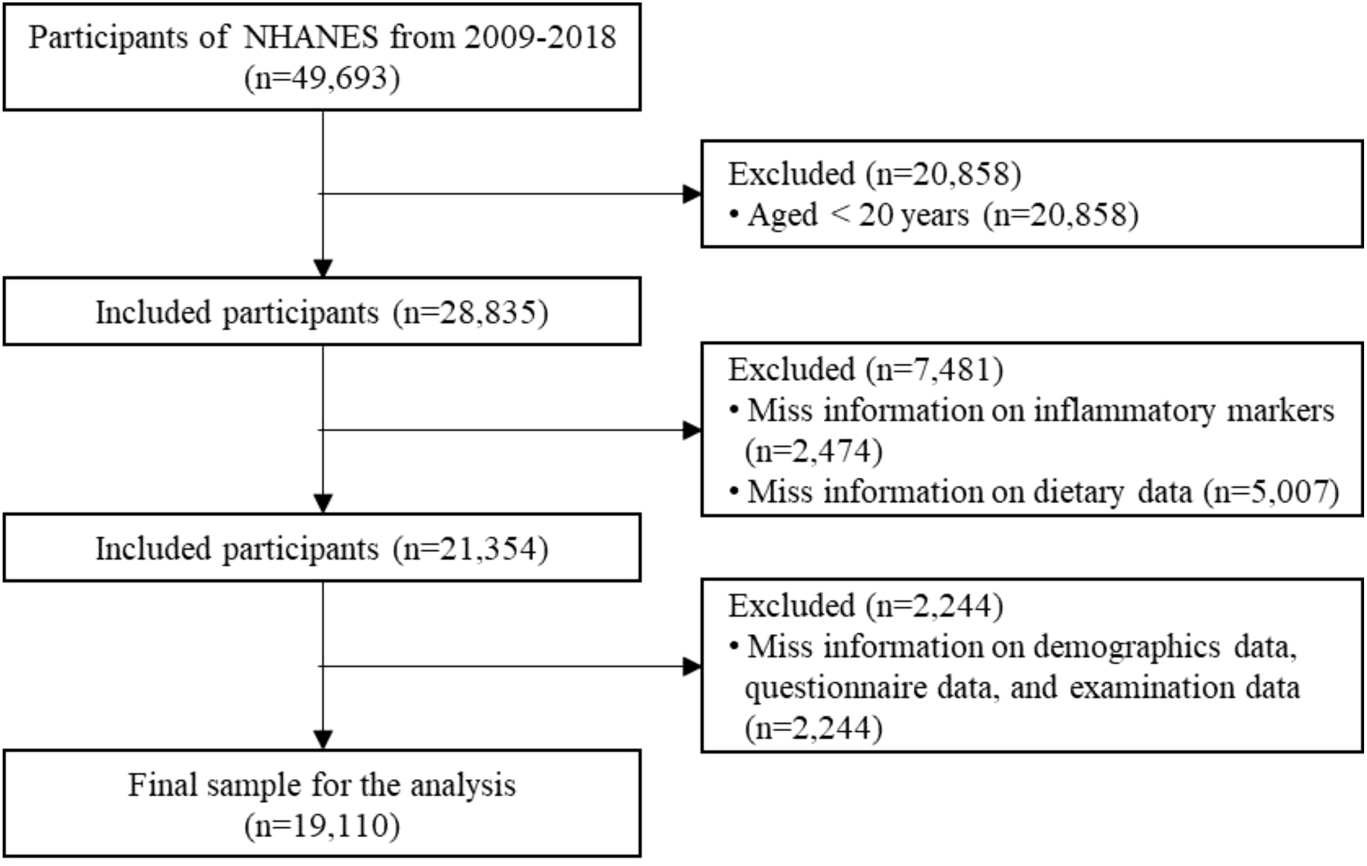
Flowchart of participant recruitment.

In this secondary data analysis, we used NHANES data from 2009 to 2018, which included a total of 49,693 participants. The following exclusion criteria were applied (1): age < 20 years; (2) missing information on inflammatory markers; (3) missing information on dietary data; (4) missing key covariate information. Finally, the study included 19,110 eligible participants. The detailed screening process is shown in **Figure 1**.

### 2.2 Assessment of diet information

In this study, two dietary indicators were calculated based on the total dietary intake data recorded in two 24-hour dietary recall interviews (both DR1TOT and DR2TOT) in NHANES. The Healthy Eating Index-2015 was used to assess the participants’ diet quality, and the Dietary Inflammatory Index was used to reflect the inflammatory potential of diet. All calculations were performed using the “Dietaryindex” package developed by Zhan et al. and the corresponding R code. “Dietaryindex” package is a flexible and validated tool that enables standardized calculations of dietary indices (28). Participants were categorized into different patterns based on their average scores across the two days.

#### 2.2.1 Healthy Eating Index-2015

We used the Healthy Eating Index-2015 (HEI-2015), developed by the National Cancer Institute (NCI) and the U.S. Department of Agriculture (USDA), to assess the participants’ overall diet quality and the quality of various dietary components (29), that can be used to assess alignment with the Dietary Guidelines for Americans (DGA). HEI-2015 consists of 13 components, including 9 adequacy components (total fruits, whole fruits, total vegetables, greens and beans, whole grains, dairy, total protein foods, seafood and plant proteins, and fatty acids) and 4 moderation components (refined grains, sodium, added sugars, and saturated fat). Adequacy components refer to dietary elements that should be consumed in greater amounts, a higher score indicates a more adequate intake. In contrast, moderation components refer to dietary elements that should be limited, a higher score indicates a lower intake and thus better alignment with dietary guidelines. The total score of HEI-2015 is the sum of the adequacy and moderation components, ranging from 0 to 100, with a higher score indicating better overall diet quality (20).

#### 2.2.2 Dietary Inflammatory Index

We used the Dietary Inflammatory Index (DII) to reflect the inflammatory potential of the diet. The DII is a dietary tool developed based on literature and population data, used to quantitatively assess the pro-inflammatory and anti-inflammatory potential of food intake. The calculation of DII involves 45 food parameters, each of which is assigned an inflammatory effect score based on its impact on six specific inflammatory markers (IL-1β, IL-4, IL-6, IL-10, TNF-α, and CRP) (21). The scores of all food parameters are then summed to obtain an individual’s DII score. The higher the DII score, the stronger the pro-inflammatory effect of the diet, and conversely, the stronger the anti-inflammatory effect. Specifically, a DII score > 0 indicates a pro-inflammatory diet, while a DII score < 0 indicates an anti-inflammatory diet. Due to the limitations of the NHANES database, our study only included 28 dietary components for DII calculations, such as energy, carbohydrates, protein, fiber, vitamin A, vitamin C, and vitamin D. Existing studies have demonstrated that the dietary components in the NHANES database basically cover the representative pro-inflammatory and anti-inflammatory nutrients, and to a certain extent, they are able to differentiate between the dietary inflammatory potential (25, 30). Moreover, the predictive power of DII for diet-related inflammation was almost unchanged when the available food parameters were reduced (31).

### 2.3 Inflammatory markers

The four inflammatory markers selected in this study were derived from the NHANES laboratory data, including white blood cells (WBC), neutrophils (Neu), neutrophil-to-lymphocyte ratio (NLR), and systemic immune-inflammation index (SII). WBC and Neu were obtained through the analysis of whole blood samples using the Beckman Coulter DxH-800 instrument at the NHANES Mobile Examination Center (MEC). NLR was obtained by calculating the ratio of neutrophil count to lymphocyte count, and SII was calculated using the following formula: SII = platelet count × neutrophil count/lymphocyte count.

### 2.4 Covariates

Based on previous studies (24, 25, 32), we identified the following potential confounding factors that may affect the study results. The specific covariates include: age (20–44 years, ≥45 years) (33, 34), gender (male, female), race (Mexican American, other Hispanic, non-Hispanic White, non-Hispanic Black, and other races), marital status (married/living with partner, widowed/divorced/separated/never married), education level (less than high school, high school graduate, above high school), family poverty income ratio (PIR < 1, 1 ≤ PIR < 2, 2 ≤ PIR < 4, PIR ≥ 4), weight status (BMI < 25 kg/m², 25 ≤ BMI < 30, BMI ≥ 30), physical activity (inactive: < 600 MET-min/week, active: 600–1200 MET-min/week, highly active: ≥ 1200 MET-min/week) (35, 36), smoking status (current, former, never) (37), drinking status (yes, no), diabetes (yes, no, and borderline), hypertension (yes, no), cardiovascular disease (CVD) (yes, no), and cancer (yes, no). Drinking status was determined based on two 24-hour dietary recall interviews, and if participants reported alcohol intake > 0 gm at least once in the 24-hour dietary recall, they were classified as alcohol consumers (38, 39). In addition, information on diabetes, hypertension, CVD (including congestive heart failure, coronary heart disease, angina, and heart attack), and cancer was obtained through self-reports in the Diabetes questionnaire, Blood pressure and cholesterol questionnaire, and Medical Conditions questionnaire.

### 2.5 Statistical analysis

This study followed the NHANES data analysis tutorial (40), adjusted the sample weights, and used the weighted sample for all statistical analyses. A new sample weight was constructed according to the NHANES analysis guidelines before analysis, by dividing the original 5-cycle sample weight by 5 (41). When describing participant characteristics, continuous variables were described by the mean ± standard deviation (SD), and categorical variables were described by number and percentage. Spearman correlation analysis was conducted to analyze the correlation between HEI-2015, DII, various inflammatory markers, and age.

This study primarily used weighted multiple linear regression models to assess the association between dietary scores (HEI-2015, DII) and inflammatory markers (WBC, Neu, NLR, SII). Three models were constructed for analysis: Model 1 was not adjusted, Model 2 adjusted for gender, age, and race, and Model 3 further adjusted for marital status, education level, family PIR, weight status, physical activity, smoking status, drinking, diabetes, hypertension, CVD, and cancer. Weighted quantile sum (WQS) regression models were used to analyze the combined effect of different HEI-2015 components and DII components on inflammatory markers. The weight of each dietary component represents its contribution to the effect of different inflammatory markers. We further divided participants into four patterns based on HEI-2015 and DII scores to explore the combined effect of dietary quality and dietary inflammatory potential on inflammatory markers: (1) Poor dietary quality and pro-inflammatory diet, (2) High dietary quality and pro-inflammatory diet, (3) Poor dietary quality and anti-inflammatory diet, (4) High dietary quality and anti-inflammatory diet. Specifically, we referred to the delineation criteria of previous studies (42–45), with HEI-2015 ≥ 50 indicating high dietary quality, HEI-2015 < 50 indicating poor dietary quality, DII > 0 indicating a pro-inflammatory diet, and DII < 0 indicating an anti-inflammatory diet.

Furthermore, in Model 3, age was included as a moderator variable and was categorized to examine its moderating effect on the associations between HEI-2015, DII, and inflammatory markers. The significance of the moderating effect was assessed by adding interaction terms (HEI-2015 × age or DII × age) in the model and using simple slope analysis to visualize the interaction terms. Statistical analyses were performed using STATA/MP version 17.0 (Stata Corp LP, College Station, TX, USA) and R software version 4.4.2 (R Foundation for Statistical Computing, Vienna, Austria). P-value < 0.05 was used to indicate statistical significance.

## 3 Results

### 3.1 Participant characteristics

**Table 1** summarizes the baseline characteristics of participants. This study included a total of 19,110 participants, comprising 10,038 females (52.53%) and 9,072 males (47.47%). Among them, 7,975 (41.73%) were aged 20–44 years, and 11,135 (58.27%) were aged 45 years or older. The average HEI-2015 score was 51.65 ± 12.09, and the average DII score was 1.09 ± 1.72.

**Table 1:** Characteristics of participants.

Characteristic	M ± SD or N (%)
**Age**	
20–44 years	7,975 (41.73)
≥45 years	11,135 (58.27)
**Gender**	
Male	9,072 (47.47)
Female	10,038 (52.53)
**Race**	
Mexican American	2,611 (13.66)
Other Hispanic	1,855 (9.71)
Non-Hispanic White	8,295 (43.41)
Non-Hispanic Black	3,982 (20.84)
Other Race^a^	2,367 (12.39)
**Education level**	
<High school	3,881 (20.31)
High school	4,280 (22.40)
>High school	10,949 (57.29)
**Marital status**	
Married/Living with partner	11,517 (60.27)
Widowed/Divorced/Separated/Never married	7,593 (39.73)
**Family PIR**	
<1	3,945 (20.64)
1-2	5,069 (26.53)
2-4	5,084 (26.60)
≥4	5,012 (26.23)
**Weight status**	
<25 kg/m^2^	5,284 (27.65)
25–30 kg/m^2^	6,137 (32.11)
≥30 kg/m^2^	7,689 (40.24)
**Physical activity**	
Inactive	7,453 (39.00)
Active	2,057 (10.76)
Highly active	9,600 (50.24)
**Smoking status**	
Current	3,628 (18.98)
Former	4,694 (24.56)
Never	10,788 (56.45)
**Drinking**	
Yes	5,509 (28.83)
No	13,601 (71.17)
**Diabetes**	
Yes	2,548 (13.33)
No	16,075 (84.12)
Borderline	487 (2.55)
**Hypertension**	
Yes	7,001 (36.64)
No	12,109 (63.36)
**CVD**	
Yes	1,565 (8.19)
No	17,545 (91.81)
**Cancer**	
Yes	1,895 (9.92)
No	17,215 (90.08)
**HEI-2015 score**	51.65 ± 12.09
**DII score**	1.09 ± 1.72
**WBC (1000 cells/uL)**	7.26 ± 3.84
**Neu (1000 cells/uL)**	4.26 ± 1.86
**NLR**	2.17 ± 1.22
**SII**	519.17 ± 385.72

CVD, cardiovascular disease; DII, Dietary Inflammatory Index; HEI-2015, Healthy Eating Index-2015; M ± SD, mean ± standard deviation; N, sample size; Neu, neutrophils; NLR, neutrophil-to-lymphocyte ratio; Other Race^a^, Other Race - Including Multi-Racial; PIR, poverty-to-income ratio; SII, systemic immune-inflammation index; WBC, white blood cells.

### 3.2 Correlation analysis of key variables

**Table 2** shows the Spearman correlation coefficients between HEI-2015, DII, inflammatory markers (WBC, Neu, NLR, SII), and age. The correlation analysis results indicated a negative correlation between HEI-2015 and DII (r = -0.5095; P < 0.001). HEI-2015 scores were negatively correlated with all four inflammatory markers (WBC, Neu, NLR, SII) (r = -0.1381, -0.1167, -0.0326, -0.0637; all P values <0.001). In contrast, DII was positively correlated with all four inflammatory markers (r = 0.0987, 0.0825, 0.0114, 0.051). Additionally, we found that age was positively correlated with HEI-2015 scores (r = 0.1529, P < 0.001), negatively correlated with WBC and Neu (r = -0.0766, -0.0433; all P values < 0.001), but positively correlated with NLR (r = 0.075; P < 0.001).

**Table 2:** Correlation analysis of HEI-2015, DII, inflammatory markers, and age.

Variables	HEI-2015	DII	WBC	Neu	NLR	SII	Age
HEI-2015	1						
DII	-0.5095^***^	1					
WBC	-0.1381^***^	0.0987^***^	1				
Neu	-0.1167^***^	0.0825^***^	0.9104^***^	1			
NLR	-0.0326^***^	0.0114	0.3552^***^	0.6733^***^	1		
SII	-0.0637^***^	0.051^***^	0.4612^***^	0.708^***^	0.8565^***^	1	
Age	0.1529^***^	0.0007	-0.0766^***^	-0.0433^***^	0.075^***^	-0.0036	1

***P < 0.001; DII, Dietary Inflammatory Index; HEI-2015, Healthy Eating Index-2015; Neu, neutrophils; NLR, neutrophil-to-lymphocyte ratio; SII, systemic immune-inflammation index; WBC, white blood cells.

### 3.3 Association between HEI-2015 and inflammatory markers

**Table 3** presents the results of the weighted multiple linear regression analysis between HEI-2015 scores and inflammatory markers. The results showed that HEI-2015 scores were significantly negatively correlated with WBC, Neu, NLR, and SII in all models, indicating that high dietary quality is associated with lower levels of the four inflammatory markers. In Model 3, which adjusted for all covariates, a 1-point increase in HEI-2015 score was associated with significant reductions in WBC (β = -0.012, 95% CI: -0.015, -0.009), Neu (β = -0.008, 95% CI: -0.010, -0.006), NLR (β = -0.002, 95% CI: -0.004, -0.001), and SII (β = -1.283, 95% CI: -1.689, -0.877). Although the impact of HEI-2015 scores on inflammatory markers slightly weakened compared to Models 1 and 2, the association between them remained unchanged.

**Table 3:** Association between HEI-2015 and inflammatory markers.

Variables	Model 1		Model 2		Model 3	
	β (95%CI)	P-value	β (95%CI)	P-value	β (95%CI)	P-value
WBC						
HEI-2015 score	-0.028 (-0.032, -0.025)	<0.001	-0.028 (-0.032, -0.025)	<0.001	-0.012 (-0.015, -0.009)	<0.001
Neu						
HEI-2015 score	-0.019 (-0.021, -0.017)	<0.001	-0.020 (-0.022, -0.018)	<0.001	-0.008 (-0.010, -0.006)	<0.001
NLR						
HEI-2015 score	-0.003 (-0.004, -0.001)	0.001	-0.004 (-0.006, -0.003)	<0.001	-0.002 (-0.004, -0.001)	0.004
SII						
HEI-2015 score	-1.621 (-1.991, -1.251)	<0.001	-2.112 (-2.473, -1.750)	<0.001	-1.283 (-1.689, -0.877)	<0.001

Model 1: no covariates were adjusted; Model 2: adjust for gender, age, race; Model 3: adjust for gender, age, race, marital status, education level, family PIR, weight status, physical activity, smoke status, drinking, diabetes, hypertension, CVD and cancer; CVD, cardiovascular disease; HEI-2015, Healthy Eating Index-2015; Neu, neutrophils; NLR, neutrophil-to-lymphocyte ratio; PIR, poverty-to-income ratio; SII, systemic immune-inflammation index; WBC, white blood cells; 95% CI, 95% confidence interval.

**Figure 2:**
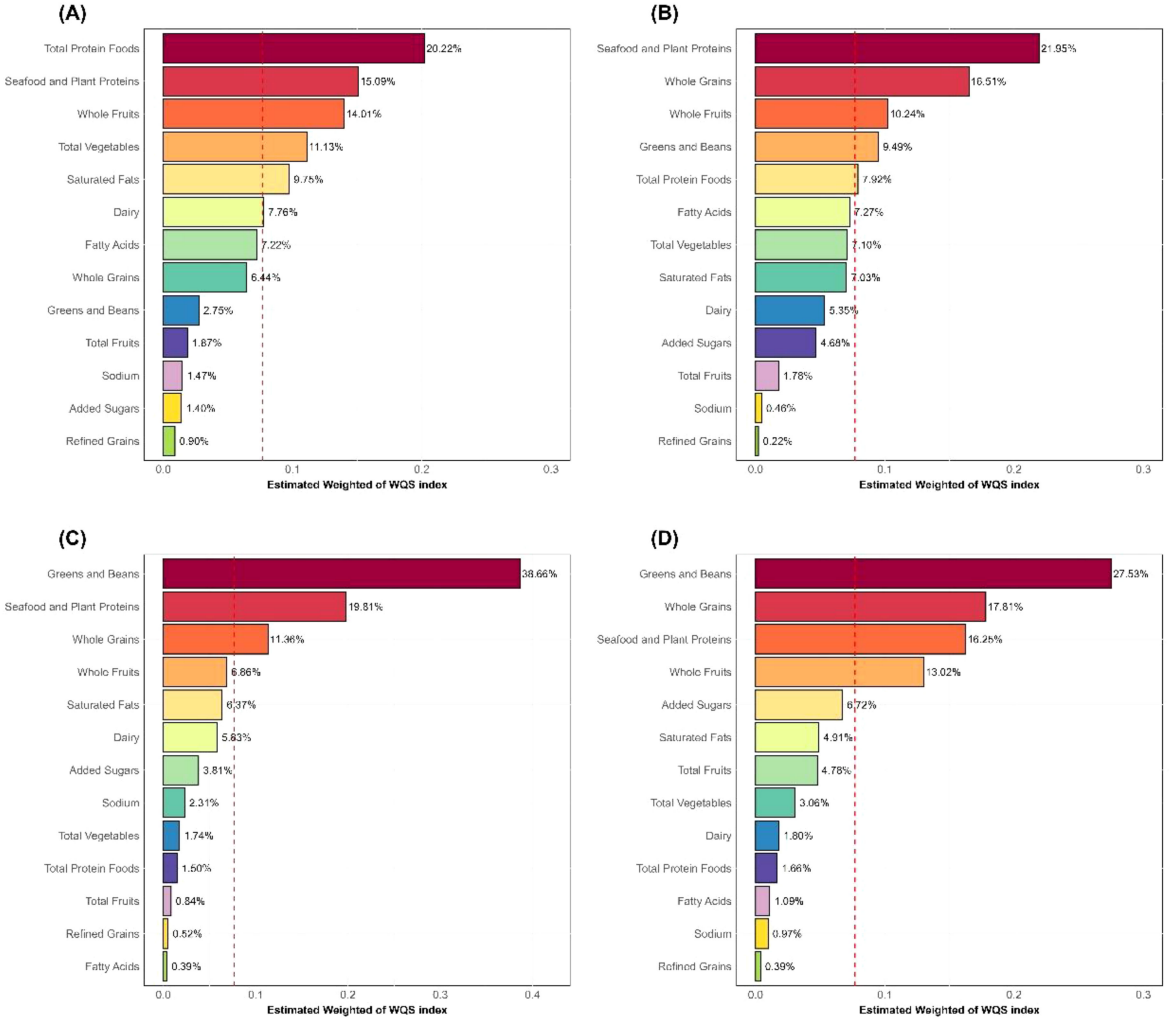
WQS regression weights of HEI-2015 components. **(A)** WBC; **(B)** Neu; **(C)** NLR; **(D)** SII; HEI-2015, Healthy Eating Index-2015; Neu, neutrophils; NLR, neutrophil-to-lymphocyte ratio; SII, systemic immune-inflammation index; WBC, white blood cells.

Furthermore, we used the WQS regression model to further explore the mixed effects of the components of HEI-2015 on inflammatory markers. Similar to the linear regression results, the WQS regression model showed that the 13 components of HEI-2015 were significantly negatively correlated with WBC (Estimate = -0.381, 95% CI: -0.541, -0.222), Neu (Estimate = -0.241, 95% CI: -0.306, -0.176), NLR (Estimate = -0.095, 95% CI: -0.146, -0.043), and SII (Estimate = -42.709, 95% CI: -57.969, -27.449), as detailed in **Supplementary Table S1**. **Figure 2** further illustrates the results of the mixed effects of HEI-2015 components on the four inflammatory markers in the WQS regression model. In the WQS-WBC model (**Figure 2A**), total protein foods, seafood and plant proteins, and whole fruits were the top three contributing dietary components, accounting for 20.22%, 15.09%, and 14.01% of the weight respectively. In the WQS-Neu model (**Figure 2B**), seafood and plant proteins (21.95%), whole grains (16.51%), and whole fruits (10.24%) were the top three components. In the WQS-NLR model (**Figure 2C**), greens and beans, seafood and plant proteins, and whole grains were the top three components, accounting for 38.66%, 19.81%, and 11.36% respectively. In the WQS-SII model (**Figure 2D**), greens and beans, whole grains, and seafood and plant proteins were the top three components, accounting for 61.59%. It is noteworthy that the top three contributing components in all four WQS models were adequacy components. In contrast, the four moderation components (refined grains, sodium, added sugars, and saturated fat) accounted for 13.52%, 12.39%, 13.01%, and 12.99% of the total weight in the WQS models, contributing relatively less to the overall results, but their role in overall dietary quality cannot be ignored.

### 3.4 Association between DII and inflammatory markers

**Table 4** presents the association between DII scores and inflammatory markers. The results indicate that DII scores were significantly positively correlated with WBC, Neu, NLR, and SII. In Model 3, which adjusted for all covariates, a 1-point increase in DII score was associated with significant increases in WBC (β = 0.057, 95% CI: 0.035, 0.080), Neu (β = 0.042, 95% CI: 0.024, 0.059), NLR (β = 0.014, 95% CI: 0.001, 0.026), and SII (β = 6.228, 95% CI: 2.521, 9.934).

**Table 4:** Association between DII and inflammatory markers.

Variables	Model 1		Model 2		Model 3	
	β (95%CI)	P-value	β (95%CI)	P-value	β (95%CI)	P-value
WBC						
DII score	0.157 (0.131, 0.183)	<0.001	0.166 (0.139, 0.192)	<0.001	0.057 (0.035, 0.080)	<0.001
Neu						
DII score	0.109 (0.090, 0.128)	<0.001	0.119 (0.100, 0.138)	<0.001	0.042 (0.024, 0.059)	<0.001
NLR						
DII score	0.013 (0.000, 0.026)	0.043	0.031 (0.019, 0.043)	<0.001	0.014 (0.001, 0.026)	0.032
SII						
DII score	12.182 (8.657, 15.707)	<0.001	12.153 (8.640, 15.666)	<0.001	6.228 (2.521, 9.934)	0.001

Model 1: no covariates were adjusted; Model 2: adjust for gender, age, race; Model 3: adjust for gender, age, race, marital status, education level, family PIR, weight status, physical activity, smoke status, drinking, diabetes, hypertension, CVD and cancer; CVD, cardiovascular disease; DII, Dietary Inflammatory Index; Neu, neutrophils; NLR, neutrophil-to-lymphocyte ratio; PIR, poverty-to-income ratio; SII, systemic immune-inflammation index; WBC, white blood cells; 95% CI, 95% confidence interval.

**Figure 3:**
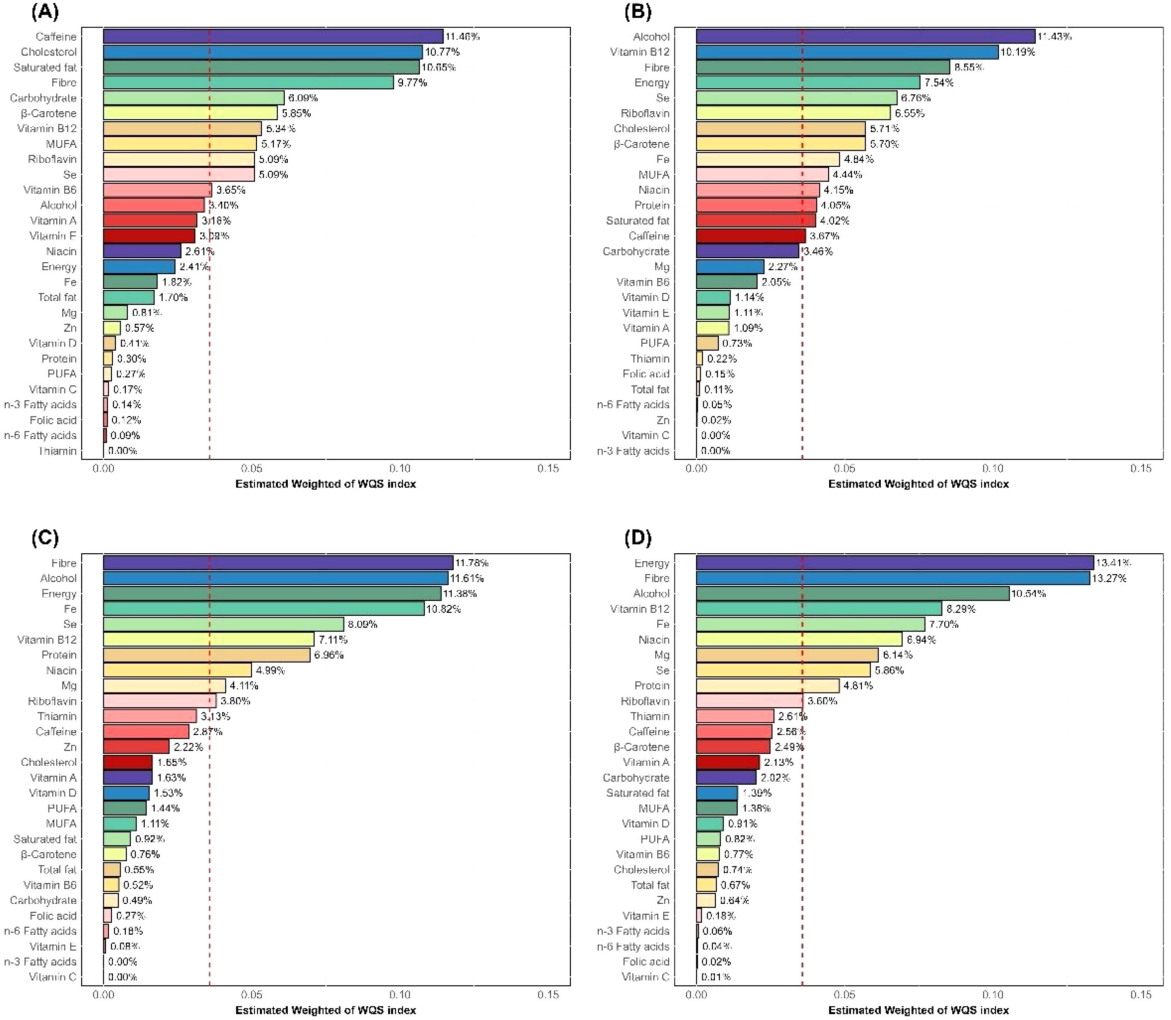
WQS regression weights of DII components. **(A)** WBC; **(B)** Neu; **(C)** NLR; **(D)** SII; DII, Dietary Inflammatory Index; Neu, neutrophils; NLR, neutrophil-to-lymphocyte ratio; SII, systemic immune-inflammation index; WBC, white blood cells.

Similarly, the results of the WQS regression model also showed that the components of DII were significantly positively correlated with WBC (Estimate = 0.470, 95% CI: 0.229, 0.711), Neu (Estimate = 0.442, 95% CI: 0.306, 0.578), NLR (Estimate = 0.207, 95% CI: 0.111, 0.304), and SII (Estimate = 70.783, 95% CI: 42.897, 98.670), as detailed in **Supplementary Table S2**. **Figure 3** illustrates the weight contributions of the DII components in the WQS model. In the WQS-WBC model (**Figure 3A**), caffeine (11.46%), cholesterol (10.77%), saturated fat (10.65%), fiber (9.77%), and carbohydrate (6.09%) were the top five contributing dietary components, almost half of the total weight. In the WQS-Neu model (**Figure 3B**), alcohol (11.43%), vitamin B12 (10.19%), fiber (8.55%), energy (7.54%), and Se (6.76%) were the top five components, accounting for 44.47% of the weight. In the WQS-NLR model (**Figure 3C**), fiber (11.78%), alcohol (11.61%), energy (11.38%), Fe (10.82%), and Se (8.09%) were the top five components, over half of total weight. In the WQS-SII model (**Figure 3D**), energy (13.41%), fiber (13.27%), alcohol (10.54%), vitamin B12 (8.29%), and Fe (7.70%) were the top five components, accounting for 53.21%. We found that fiber was the most frequently occurring component among the top five contributors, followed by alcohol and energy.

### 3.5 Joint association of HEI-2015 and DII with inflammatory markers

**Table 5** presents the combined effect of HEI-2015 and DII on different inflammatory markers. In model 1, compared to pattern 1 (poor dietary quality and pro-inflammatory diet), pattern 2 (high dietary quality and pro-inflammatory diet), pattern 3 (poor dietary quality and anti-inflammatory diet), and pattern 4 (high dietary quality and anti-inflammatory diet) were all significantly associated with lower levels of WBC, Neu, and SII. However, only pattern 4 was significantly associated with lower NLR, while the associations between other patterns and NLR were not statistically significant (P > 0.05). Similarly, in Model 3, pattern 4 was significantly associated with lower levels of WBC (β = -0.293, 95% CI: -0.399, -0.187), Neu (β = -0.226, 95% CI: -0.309, -0.142), NLR (β = -0.084, 95% CI: -0.146, -0.023), and SII (β = -34.381, 95% CI: -52.113, -16.648). Although pattern 2 did not significantly affect NLR (P = 0.051), it still significantly reduced WBC (β = -0.228, 95% CI: -0.333, -0.123), Neu (β = -0.154, 95% CI: -0.226, -0.081), and SII (β = -25.244, 95% CI: -37.398, -13.091), which had slightly lower effects than pattern 4. However, the associations between pattern 3 and all four inflammatory markers were not statistically significant. Considering the results from all three models, pattern 4 showed the greatest benefit, whereas pattern 3 showed not significant improvement. These results suggest that improving dietary quality can mitigate the negative effects of pro-inflammatory diet on inflammatory markers, while anti-inflammatory diet alone is insufficient to counteract the impact of poor dietary quality on inflammatory markers.

**Table 5:** Joint association of HEI-2015 and DII with inflammatory markers.

Variables	Model 1		Model 2		Model 3	
	β (95%CI)	P-value	β (95%CI)	P-value	β (95%CI)	P-value
WBC						
Pattern 1	Ref		Ref		Ref	
Pattern 2	-0.482 (-0.594, -0.369)	<0.001	-0.481 (-0.592, -0.371)	<0.001	-0.228 (-0.333, -0.123)	<0.001
Pattern 3	-0.340 (-0.517, -0.163)	<0.001	-0.342 (-0.525, -0.160)	<0.001	-0.134 (-0.298, 0.029)	0.106
Pattern 4	-0.756 (-0.881, -0.630)	<0.001	-0.757 (-0.881, -0.633)	<0.001	-0.293 (-0.399, -0.187)	<0.001
Neu						
Pattern 1	Ref		Ref		Ref	
Pattern 2	-0.316 (-0.395, -0.236)	<0.001	-0.332 (-0.407, -0.256)	<0.001	-0.154 (-0.226, -0.081)	<0.001
Pattern 3	-0.234 (-0.369, -0.099)	0.001	-0.237 (-0.372, -0.101)	0.001	-0.086 (-0.212, 0.039)	0.174
Pattern 4	-0.531 (-0.619, -0.443)	<0.001	-0.554 (-0.641, -0.467)	<0.001	-0.226 (-0.309, -0.142)	<0.001
NLR						
Pattern 1	Ref		Ref		Ref	
Pattern 2	-0.042 (-0.084, 0.001)	0.053	-0.066 (-0.108, -0.024)	0.002	-0.046 (-0.092, 0.000)	0.051
Pattern 3	-0.006 (-0.107, 0.095)	0.904	-0.047 (-0.145, 0.051)	0.341	-0.009 (-0.109, 0.091)	0.857
Pattern 4	-0.083 (-0.143, -0.022)	0.008	-0.145 (-0.204, -0.086)	<0.001	-0.084 (-0.146, -0.023)	0.008
SII						
Pattern 1	Ref		Ref		Ref	
Pattern 2	-26.331 (-37.885, -14.778)	<0.001	-36.717 (-47.876, -25.558)	<0.001	-25.244 (-37.398, -13.091)	<0.001
Pattern 3	-27.628 (-54.971, -0.284)	0.048	-19.378 (-46.652, 7.895)	0.161	-7.123 (-35.154, 20.908)	0.614
Pattern 4	-52.599 (-68.853, -36.345)	<0.001	-58.452 (-74.787, -42.118)	<0.001	-34.381 (-52.113, -16.648)	<0.001

Model 1: no covariates were adjusted; Model 2: adjust for gender, age, race; Model 3: adjust for gender, age, race, marital status, education level, family PIR, weight status, physical activity, smoke status, drinking, diabetes, hypertension, CVD and cancer; Pattern 1: poor dietary quality and pro-inflammatory diet; Pattern 2: high dietary quality and pro-inflammatory diet; Pattern 3: poor dietary quality and anti-inflammatory diet; Pattern 4: high dietary quality and anti-inflammatory diet; CVD, cardiovascular disease; Neu, neutrophils; NLR, neutrophil-to-lymphocyte ratio; PIR, poverty-to-income ratio; SII, systemic immune-inflammation index; WBC, white blood cells; 95% CI, 95% confidence interval.

### 3.6 Moderating effect of age on dietary score and inflammatory markers

To explore whether the association between different dietary scores and inflammatory markers varies by age, this study divided participants into younger adults (20–44 years) and older adults (≥45 years) groups based on age. The interaction terms between dietary scores (HEI-2015 and DII) and age were included based on Model 3, and the results are shown in **Table 6**. The results showed that in Model 4, the interaction term between HEI-2015 and age had a significant negative effect on both WBC (β = -0.008, 95% CI: -0.015, -0.001, P = 0.036), and Neu (β = -0.006, 95% CI: -0.010, -0.001, P = 0.026). This result indicates that age significantly enhances the association between HEI-2015 and WBC, and Neu. The effect of higher HEI-2015 scores to increase levels of WBC and Neu was more pronounced in the older adults. Additionally, the interaction term between DII and age had a significant negative effect on SII (β = -5.965, 95% CI: -11.460, -0.470, P = 0.034), suggesting that age significantly suppresses the association between DII and SII. The effect of higher DII scores to increase SII levels was more pronounced in the younger adults.

**Table 6:** Results for the effects of the interaction on inflammatory markers.

Variables	Model 3		Model 4	
	β (95%CI)	P-value	β (95%CI)	P-value
WBC				
HEI-2015 score	-0.012 (-0.015, -0.009)	<0.001	-0.008 (-0.012, -0.003)	0.001
Age	-0.502 (-0.593, -0.411)	<0.001	-0.505 (-0.596, -0.413)	<0.001
HEI-2015 score * Age	–	–	**-0.008 (-0.015, -0.001)**	**0.036**
DII score	0.057 (0.035, 0.080)	<0.001	0.069 (0.037, 0.100)	<0.001
Age	-0.536 (-0.627, -0.446)	<0.001	-0.539 (-0.630, -0.449)	<0.001
DII score * Age	–	–	-0.020 (-0.069, 0.029)	0.424
Neu				
HEI-2015 score	-0.008 (-0.010, -0.006)	<0.001	-0.005 (-0.008, -0.002)	0.002
Age	-0.305 (-0.376, -0.235)	<0.001	-0.308 (-0.378, -0.237)	<0.001
HEI-2015 score * Age	–	–	**-0.006 (-0.010, -0.001)**	**0.026**
DII score	0.042 (0.024, 0.059)	<0.001	0.055 (0.032, 0.078)	<0.001
Age	-0.329 (-0.400, -0.259)	<0.001	-0.333 (-0.403, -0.263)	<0.001
DII score * Age	–	–	-0.024 (-0.058, 0.010)	0.162
NLR				
HEI-2015 score	-0.002 (-0.004, -0.001)	0.004	-0.001 (-0.003, 0.001)	0.183
Age	0.094 (0.052, 0.136)	<0.001	0.093 (0.051, 0.135)	<0.001
HEI-2015 score * Age	–	–	-0.002 (-0.005, 0.001)	0.283
DII score	0.014 (0.001, 0.026)	0.032	0.025 (0.011, 0.040)	0.001
Age	0.088 (0.045, 0.130)	<0.001	0.085 (0.042, 0.127)	<0.001
DII score * Age	–	–	-0.020 (-0.043, 0.002)	0.074
SII				
HEI-2015 score	-1.283 (-1.689, -0.877)	<0.001	-0.882 (-1.427, -0.337)	0.002
Age	1.589 (-11.390, 14.568)	0.808	1.314 (-11.724, 14.353)	0.841
HEI-2015 score * Age	–	–	-0.696 (-1.474, 0.083)	0.079
DII score	6.228 (2.521, 9.934)	0.001	9.603 (5.510, 13.695)	<0.001
Age	-2.069 (-15.112, 10.974)	0.753	-2.967 (-16.142, 10.207)	0.655
DII score * Age	–	–	**-5.965 (-11.460, -0.470)**	**0.034**

Model 3: adjust for gender, age, race, marital status, education level, family PIR, weight status, physical activity, smoke status, drinking, diabetes, hypertension, CVD, and cancer; Model 4: adjusted for Model 3 plus the interaction term between dietary score and age; CVD, cardiovascular disease; DII, Dietary Inflammatory Index; HEI-2015, Healthy Eating Index-2015; Neu, neutrophils; NLR, neutrophil-to-lymphocyte ratio; PIR, poverty-to-income ratio; SII, systemic immune-inflammation index; WBC, white blood cells; 95% CI, 95% confidence interval; bold values indicate statistically significant interaction terms (P < 0.05).

Simple slope analysis further revealed that, compared to the younger adults, the slopes of the association between HEI-2015 and WBC, Neu were steeper in the older adults, as detailed in **Supplementary Figures S1**, **S2**. This indicates that the reduction effects of HEI-2015 on WBC and Neu were stronger in the older adults than in the younger adults. Conversely, the slope of the association between DII and SII was flatter in the older adults compared to the younger adults, as shown in **Supplementary Figure S3**. This suggests that the association between DII and SII is weaker in the older adults than in the younger adults.

## 4 Discussion

In this large-scale study, data from five NHANES cycles were used to systematically investigate the associations between two dietary scores (HEI-2015 and DII) and four inflammatory markers (WBC, Neu, NLR, and SII). The results revealed that high HEI-2015 scores and low DII scores were both associated with reduced levels of inflammatory markers. Notably, joint effect analysis demonstrated that high dietary quality might mitigate the adverse effects of pro-inflammatory diets on inflammatory markers, whereas an anti-inflammatory diet alone was insufficient to counteract the negative impacts of poor dietary quality. Furthermore, we examined the moderating effect of age on the association between dietary scores and inflammatory markers.

Firstly, the HEI-2015 score was used to assess individual dietary quality. In this study, a significant negative correlation was observed between HEI-2015 scores and levels of WBC, Neu, NLR, and SII among U.S. adults, indicating that high HEI-2015 scores were significantly associated with reduced inflammatory marker levels, consistent with previous research. A case-control study in an Iranian population demonstrated significant inverse correlations between HEI-2015 scores and multiple inflammatory markers, including IL-4, IL-1β, and hs-CRP (46). Similarly, another case-control study found that higher Mediterranean diet adherence score (MDS) and HEI-2010 scores were associated with lower inflammatory marker levels, thereby reducing the risk of COVID-19 infection (47). In an observational trial targeting healthy adults, HEI-2015 scores were negatively correlated with gastrointestinal inflammation markers, such as calprotectin (48). Meanwhile, WQS regression analysis was conducted to further elucidate the contributions of the 13 HEI-2015 dietary components to different inflammatory markers. The comprehensive analysis found that seafood and plant protein, whole grains, greens and beans, whole fruits, and total protein foods had a greater impact on HEI-2015 scores, which was supported by prior studies. A randomized clinical trial showed that a non-soya legume-based therapeutic lifestyle change (TLC) diet significantly reduced inflammatory markers (hs-CRP, IL-6, TNF-α) in overweight diabetic patients (49). Similarly, research on the UK Biobank database revealed that a prudent diet abundant in whole grains, vegetables, fruits, and fish was negatively correlated with most inflammatory markers (50). Another randomized controlled trial found that compared to refined grains, whole-grain consumption significantly reduced circulating levels of pro-inflammatory cytokines (IL-22 and IL-23), which were associated with optimized short-chain fatty acid profiles and changes in CD4^+^T cell distributions (51). Tomato extract, lutein, and lycopene exert anti-inflammatory properties via NF-κB signaling pathway inhibition (52). In daily life, a high-quality diet usually contains seafood, greens, fruits, and nuts, which are rich in antioxidants and anti-inflammatory nutrients (53–55). These components can not only reduce the inflammatory response, but also inhibit the synthesis of pro-inflammatory cytokines and promote the production of anti-inflammatory mediators. For example, Omega-3 polyunsaturated fatty acids, which are rich in seafood, serve as bioactive molecules, influencing the function of immune cells and exhibiting anti-inflammatory effects (56). However, low-quality diets often contain high sugar, trans fats and additives, which can promote the activation of inflammatory pathways and increase the levels of inflammatory markers (57). For example, high-sugar diets can induce inflammation by increasing blood glucose and insulin levels (58). In addition, there is a certain association between diet quality and food processing. Ultraprocessed foods, which are popular in the U.S., may reduce diet quality to some extent due to higher intake of energy-dense foods with added sugar and lower consumption of dietary fiber, with potential effects on health outcomes (59). Moreover, the processing methods themselves (such as high-temperature frying or the addition of emulsifiers) may disrupt metabolic homeostasis and consequently exacerbate inflammation. Therefore, adherence to a high-quality diet should be maintained to reduce the potential risk of inflammation and thereby improve health status.

Secondly, the DII score was employed to evaluate the inflammatory potential of individual diets. The study identified positive correlations between DII scores and these four inflammatory markers, suggesting that high DII scores were associated with elevated inflammatory marker levels. A cross-sectional analysis based on the Moli-Sani study reported positive associations between DII scores and each inflammatory biomarker in the INFLA-score (including CRP, WBC, and NLR) (60). Similarly, research from the European Prospective Investigation into Cancer and Nutrition (EPIC) cohort found positive associations between the four dietary inflammatory scores and multiple inflammatory biomarkers (32). WQS regression analyses showed that fiber, caffeine, alcohol, energy, vitamin B12, Fe, Se, and cholesterol in DII were the dietary components that primarily influenced the four inflammatory markers. Notably, although fiber, caffeine, and alcohol, which had high weights, are anti-inflammatory components, their insufficient intake may limit the ability of the DII score to reflect the anti-inflammatory potential of the overall diet. Overall, this study not only confirms the critical roles of high-quality and anti-inflammatory diets in reducing inflammation but also highlights the differential contributions of dietary components, offering insights for developing precise dietary interventions.

Additionally, the moderating effects of age were explored on the association between dietary scores and inflammatory markers. The results suggested that age exhibited a positive moderating effect on the association between HEI-2015 and WBC/Neu, while it showed a negative moderating effect on the association between DII and SII. This implies that age enhances the anti-inflammatory benefits of high-quality diets while diminishing the promotive effects of pro-inflammatory diets on inflammatory levels. Age-related physiological changes, such as redox imbalance, immunosenescence, and coagulation activation, may exacerbate inflammation. Thus, elderly tailored dietary patterns can contribute to reducing inflammation and age-related disease risks (61, 62). During this process, adopting an appropriate dietary pattern proves particularly crucial. A high-quality diet abundant in essential nutrients such as premium proteins, multiple vitamins, and fiber can provide comprehensive nutritional support for the organism, thereby effectively reducing levels of inflammatory markers. In this study, the steeper slope of HEI-2015 associations with inflammatory markers in the older adults further emphasized the importance of dietary quality in preventing inflammation-related diseases in these populations. Conversely, although pro-inflammatory diets are generally considered to exacerbate inflammatory levels, the association between DII and SII was attenuated in the older adults in this study. However, this should not be interpreted as negating the health risks of pro-inflammatory diets for the elderly population, as long-term adherence to such dietary patterns may still exert detrimental effects on the organism. These findings indicate that age is not merely a confounding factor but a critical moderator, emphasizing the need for age-specific dietary interventions.

Previous studies only focused on the potential effects of HEI-2015 or DII on inflammatory markers and health outcomes, ignoring the synergistic or antagonistic effects of different dietary characteristics on inflammatory response. This study innovatively explored the association between diet and inflammation from the two dimensions of dietary quality and dietary inflammatory potential. Based on the HEI-2015 scores and DII scores, four differentiated dietary patterns were divided and analyzed. The results of the joint analysis showed that after adjusting covariates, individuals with both high dietary quality and anti-inflammatory diets (Pattern 4) exhibited significantly lower levels of all inflammatory markers (WBC, Neu, NLR, SII), suggesting amplified benefits from synergy. Notably, even though individuals were on pro-inflammatory diets, sufficiently high dietary quality could partially offset the adverse effects. (Pattern 2). However, adhering to anti-inflammatory diets without improving overall dietary quality (Pattern 3) showed no statistically significant associations with inflammatory markers. This indicated that for individuals with poor dietary quality, anti-inflammatory diets alone may fail to counteract the detrimental impacts of it on immune homeostasis. Overall, the association between dietary patterns and inflammatory markers is complex and synergic. A high dietary quality could mitigate the adverse effects of pro-inflammatory diets. Importantly, concurrent improvement in overall dietary quality and reduction of pro-inflammatory components play a critical role in lowering systemic inflammation and promoting health outcomes. Furthermore, the innovative joint analysis provides a new perspective for in-depth understanding of the complex association between diet and inflammation, and establishes the foundation for the development of personalized anti-inflammatory diet intervention strategies.

This study has several limitations. First, research targets only covered U.S. adults, but dietary habits may vary across different regions, ethnicities, and cultures, which could potentially influence the study results and limit the generalization and universality of the findings. Therefore, it is recommended that future studies include more diverse populations to validate the associations observed in this study. Second, total nutrient intake data were obtained from two 24-hour dietary recall interviews, which may introduce recall bias. Third, dietary quality was assessed using the HEI-2015, which does not account for factors such as meal timing and total dietary intake. Fourth, the DII score was using limited dietary components due to database constraints, and considering the lack of comprehensive and detailed food component data from dietary supplements, we excluded this portion of the data, which may underestimate the intake of certain nutrients. Future studies should include a wider range of dietary components to calculate DII scores to further clarify the complex associations between dietary patterns and inflammatory states. Fifth, although numerous covariates were adjusted for, the possibility of residual or unknown confounding cannot be ruled out due to the numerous potential determinants influencing inflammatory markers and inherent database limitations. Therefore, we suggest that potential influencing factors such as medical and work-related factors, be considered extensively in future research. Finally, the cross-sectional design used in this study precluded causal inferences, further longitudinal studies and robustness analysis are indispensable to validate causal associations between dietary and inflammatory markers.

## 5 Conclusion

To conclude, our findings demonstrated significant negative correlations between HEI-2015 and levels of WBC, Neu, NLR, and SII, whereas DII scores showed positive correlations with all four inflammatory markers. It was worth noting that age played a critical moderating role in these associations. Moreover, high dietary quality could mitigate the adverse effects of pro-inflammatory diets, whereas anti-inflammatory diets alone could not counteract the impacts of poor dietary quality. Synergistic benefits emerged when combining high dietary quality with anti-inflammatory practices. These findings offer scientific rationale for dietary strategies to reduce inflammation, however, as the study employs a cross-sectional design, it cannot establish causality.

## Data Availability

Publicly available datasets were analyzed in this study. This data can be found here: https://www.cdc.gov/nchs/nhanes/index.html.
