# Supplementary material for: Association of dietary quality and dietary inflammatory potential with inflammatory markers: evidence from the national health and nutrition examination survey 2009-2018
Source: Front Immunol. 2025 Jun 5;16:1596806. doi: 10.3389/fimmu.2025.1596806 (PMC12176585; doi:10.3389/fimmu.2025.1596806)
Supplement: Supplementary file 1 [file DataSheet1.docx]

Supplementary Material

**Supplementary Table S1.** The results of the WQS regression model for the association between HEI-2015 components and inflammatory markers

| **Inflammatory markers** | **Model 1** | | **Model 2** | | **Model 3** | |
| --- | --- | --- | --- | --- | --- | --- |
|  | **Estimate (95%CI)** | **P-value** | **Estimate (95%CI)** | **P-value** | **Estimate (95%CI)** | **P-value** |
| **WBC** | -0.607 (-0.738, -0.476) | ＜0.001 | -0.675 (-0.815, -0.535) | ＜0.001 | -0.381 (-0.541, -0.222) | ＜0.001 |
| **Neu** | -0.407 (-0.467, -0.347) | ＜0.001 | -0.447 (-0.509, -0.386) | ＜0.001 | -0.241 (-0.306, -0.176) | ＜0.001 |
| **NLR** | -0.145 (-0.197, -0.094) | ＜0.001 | -0.143 (-0.194, -0.091) | ＜0.001 | -0.095 (-0.146, -0.043) | ＜0.001 |
| **SII** | -53.160 (-69.132, -37.188) | ＜0.001 | -56.189 (-71.024, -41.353) | ＜0.001 | -42.709 (-57.969, -27.449) | ＜0.001 |

Model 1: no covariates were adjusted; Model 2: adjust for gender, age, race; Model 3: adjust for gender, age, race, marital status, education level, family PIR, weight status, physical activity, smoke status, drinking, diabetes, hypertension, CVD and cancer; CVD, cardiovascular disease; Neu, neutrophils; NLR, neutrophil-to-lymphocyte ratio; PIR, poverty-to-income ratio; SII, systemic immune-inflammation index; WBC, white blood cells; 95% CI, 95% confidence interval

**Supplementary Table S2.** The results of the WQS regression model for the association between DII components and inflammatory markers

| **Inflammatory markers** | **Model 1** | | **Model 2** | | **Model 3** | |
| --- | --- | --- | --- | --- | --- | --- |
|  | **Estimate (95%CI)** | **P-value** | **Estimate (95%CI)** | **P-value** | **Estimate (95%CI)** | **P-value** |
| **WBC** | 1.000 (0.789, 1.210) | ＜0.001 | 1.018 (0.800, 1.236) | ＜0.001 | 0.470 (0.229, 0.711) | ＜0.001 |
| **Neu** | 0.787 (0.668, 0.907) | ＜0.001 | 0.832 (0.712, 0.952) | ＜0.001 | 0.442 (0.306, 0.578) | ＜0.001 |
| **NLR** | 0.232 (0.153, 0.311) | ＜0.001 | 0.290 (0.201, 0.380) | ＜0.001 | 0.207 (0.111, 0.304) | ＜0.001 |
| **SII** | 81.880 (57.796, 105.963) | ＜0.001 | 95.064 (69.002, 121.126) | ＜0.001 | 70.783 (42.897, 98.670) | ＜0.001 |

Model 1: no covariates were adjusted; Model 2: adjust for gender, age, race; Model 3: adjust for gender, age, race, marital status, education level, family PIR, weight status, physical activity, smoke status, drinking, diabetes, hypertension, CVD and cancer; CVD, cardiovascular disease; Neu, neutrophils; NLR, neutrophil-to-lymphocyte ratio; PIR, poverty-to-income ratio; SII, systemic immune-inflammation index; WBC, white blood cells; 95% CI, 95% confidence interval

**
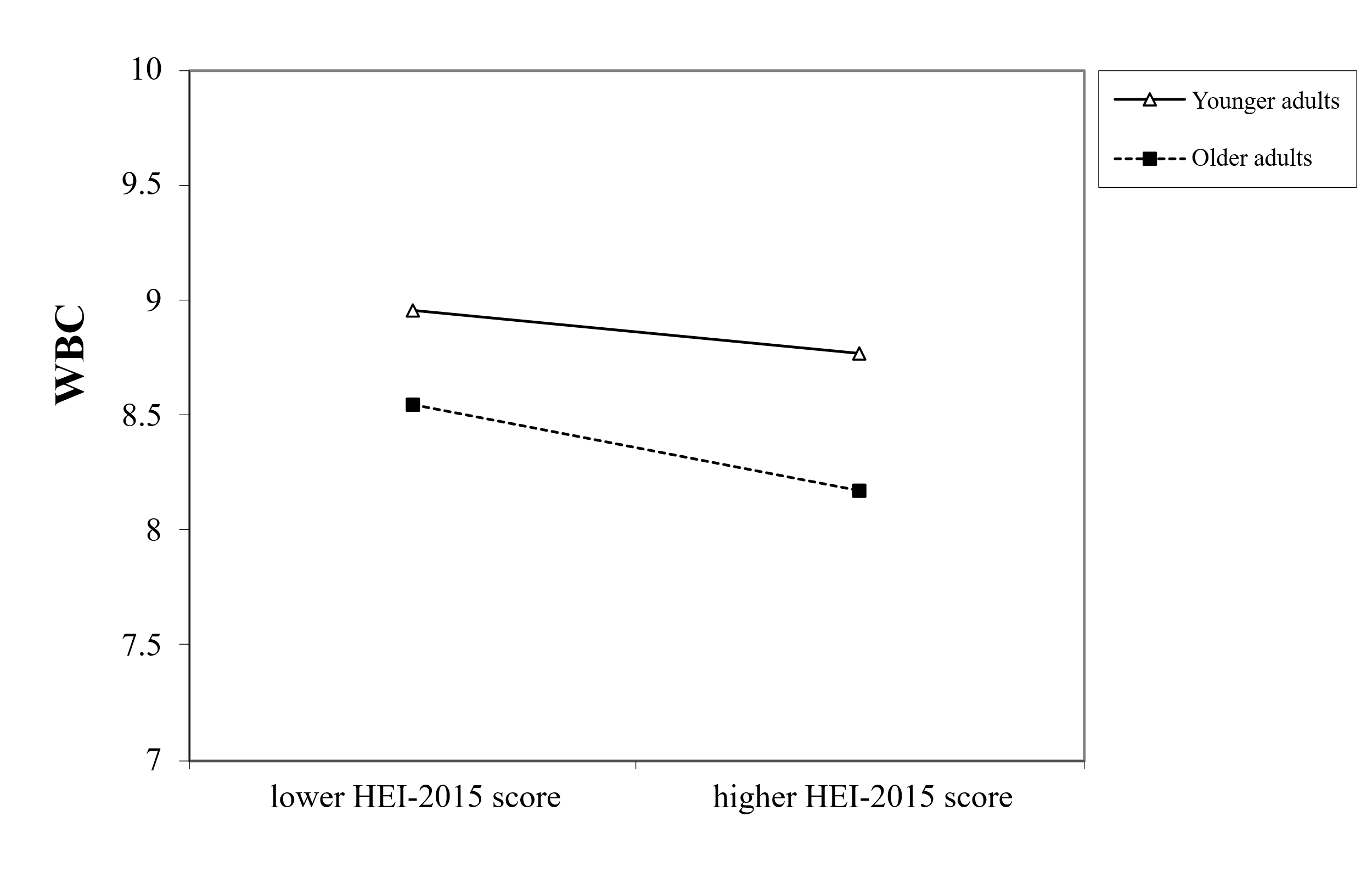
**

**Supplementary Figure S1.** The Moderating effect of age on the association between HEI-2015 score and WBC

HEI-2015, Healthy Eating Index-2015; WBC, white blood cells

**
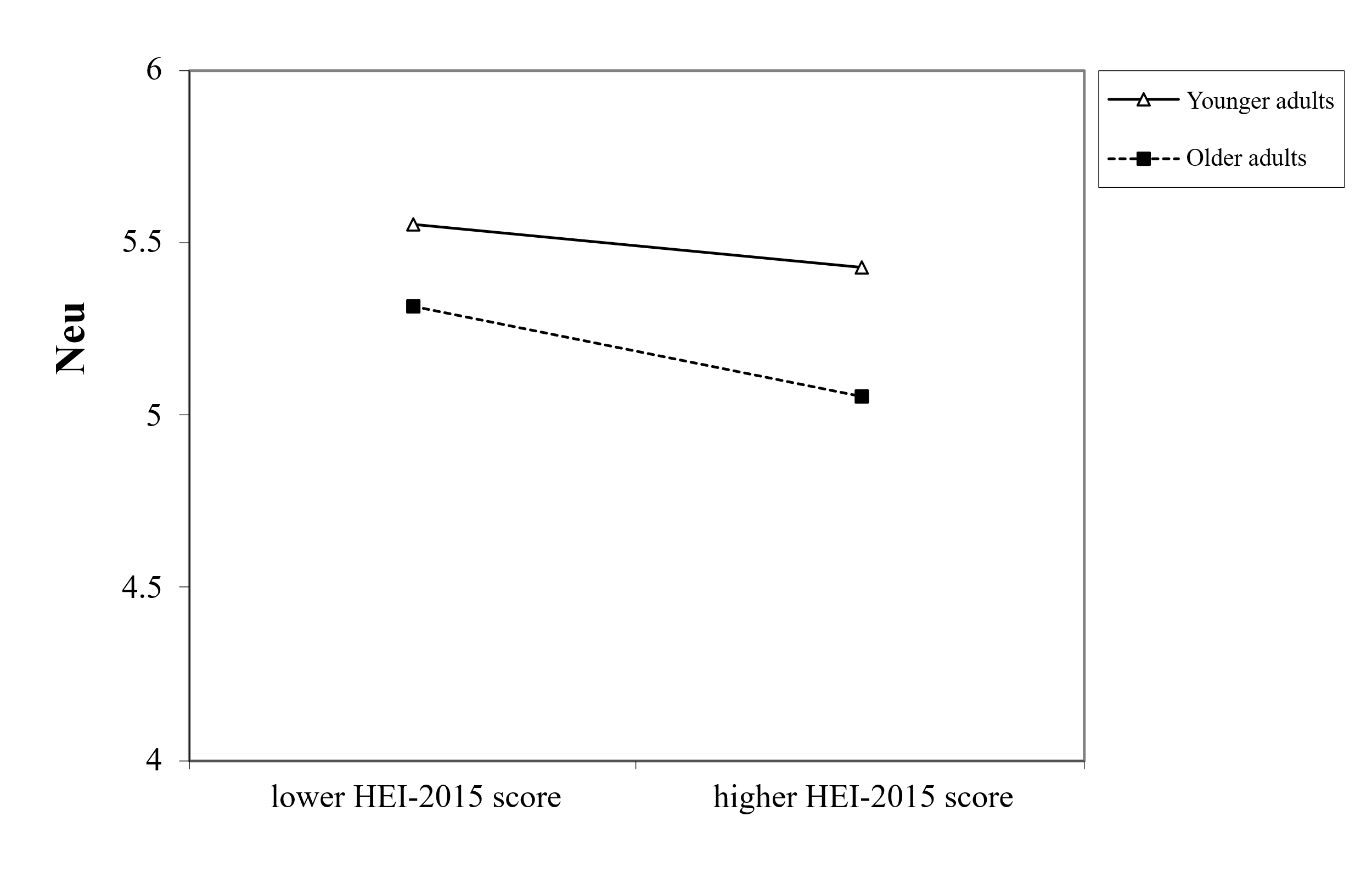
**

**Supplementary Figure S2.** The Moderating effect of age on the association between HEI-2015 score and Neu

HEI-2015, Healthy Eating Index-2015; Neu, neutrophils

**
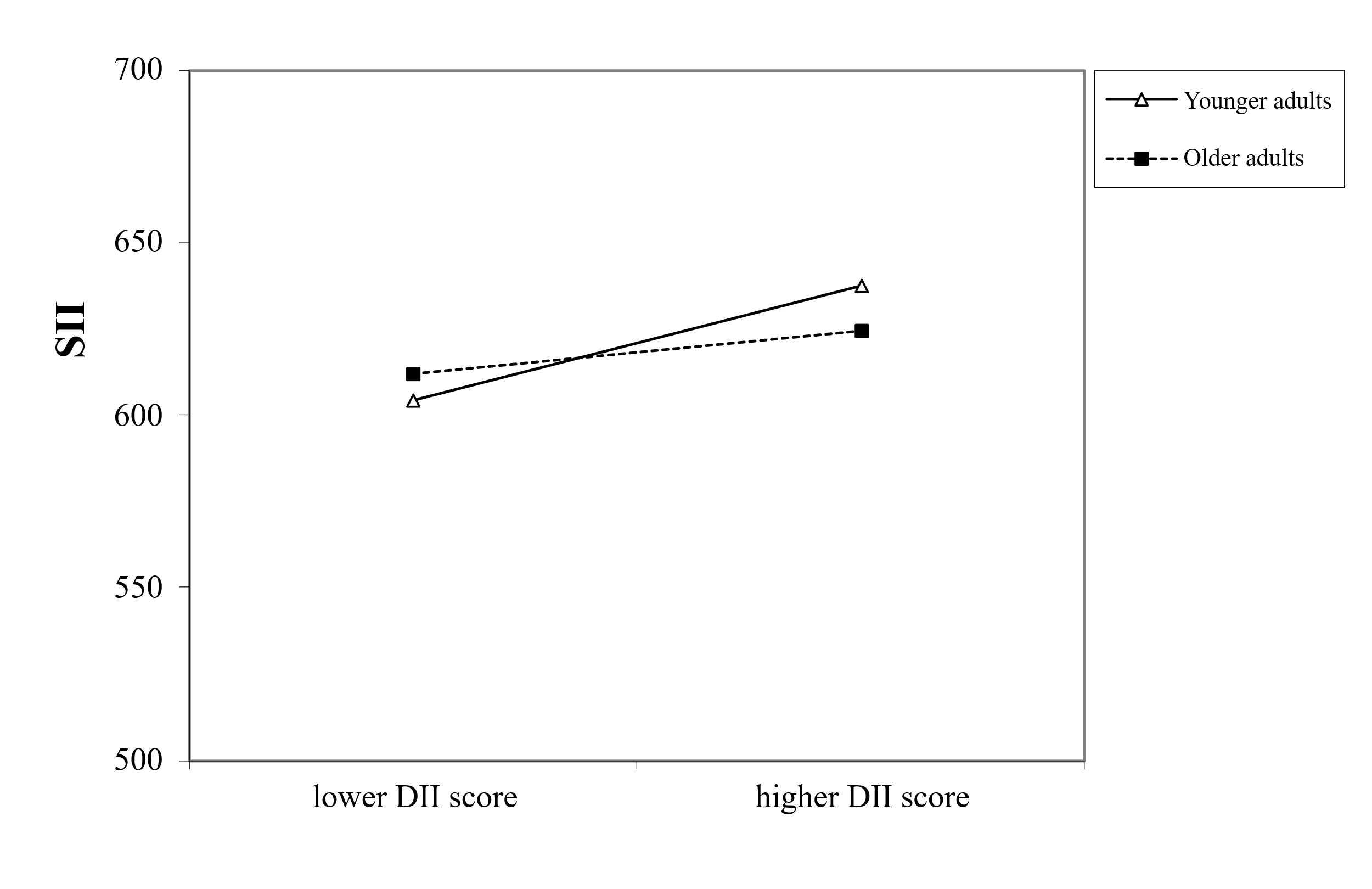
**

**Supplementary Figure S3.** The Moderating effect of age on the association between DII score and SII

DII, Dietary Inflammatory Index; SII, systemic immune-inflammation index
